# Doggy doctorates: Early career progression outcomes for UK‐funded postgraduate canine health and welfare researchers

**DOI:** 10.1002/vro2.70048

**Published:** 2026-10-14

**Authors:** Alison M. Skipper, Rowena M. A. Packer, Dan G. O'Neill

**Affiliations:** ^1^ Department of Pathobiology and Population Sciences The Royal Veterinary College Hatfield UK; ^2^ Department of Clinical Science and Services The Royal Veterinary College Hatfield UK

## Abstract

**Background:**

This quantitative study describes characteristics and career outcomes (in August 2024) of students completing research master's or PhD degrees in canine health and welfare who received not‐for‐profit funding between 2012 and 2018.

**Methods:**

Historical canine health and welfare research funding data were used to identify research students whose subsequent career outcomes were established by visual scrutiny of public online databases. Data were anonymised, aggregated and analysed to determine overall career patterns.

**Results:**

This cohort (*n* = 50) comprised 23 veterinarians and 27 non‐veterinarians, with an approximately 4:1 female:male ratio. In 2024, 23 (50.0%) of the traceable prior students had secured a permanent job, but 63.0% of the prior students employed within academia were significantly less likely to have a permanent role than those working outside academia (*p* = 0.002). All those employed remained within the science and health sector, and the majority (71.7%) worked in animal‐focused roles.

**Limitations:**

This study is retrospective and quantitative and considers only the canine sector.

**Conclusion:**

Postgraduate research degrees in canine health and welfare are undertaken by some veterinarians and non‐veterinarians and lead to varied subsequent employment opportunities. However, these results support wider concerns that early career pathways in academia provide less secure employment opportunities than comparable alternatives.

## INTRODUCTION

The UK veterinary sector currently faces many difficulties.^1^ Recent investigation by the Royal College of Veterinary Surgeons (RCVS) has confirmed an increasing trend for veterinary surgeons to leave the practising register. Concerningly, many of these leavers are young (53% were <35 in 2021), and the majority cite work dissatisfaction (poor work‒life balance, not feeling valued) as contributory reasons for their departure.^1^ Within that report, veterinary surgeons with a postgraduate qualification were less likely to leave the practising register than those without, suggesting that upskilling can improve workplace satisfaction (although it is also possible that those who are less dissatisfied are more likely to invest in upskilling). These RCVS data do not differentiate between postgraduate clinical certificates and specialty training, which would generally advance a career in clinical practice, and academic postgraduate degrees, which would potentially lead to a career in academia (a term used here to describe work in university‐based research and/or teaching). However, developing an academic career offers one potential pathway for a veterinary professional seeking an alternative to primary care practice. An academic career could also be seen as an attractive option for non‐veterinarians who desire an intellectually challenging animal‐based role.

However, there is ample evidence that establishing an academic career can also be a difficult process, with limited funding opportunities, lack of sector transparency, academic precarity, inequality of access, financial uncertainty and other difficulties all regularly described as global issues for new entrants across the academic sector, from the humanities and social sciences^2^ to the various science, technology, engineering and medicine (STEM) subjects.^3^, ^4^, ^5^, ^6^ Even 10 years ago, approximately 70% of all British PhD graduates had left academia by 3.5 years after graduation: it is often difficult to obtain postdoctoral fixed‐term contracts, and even harder to transition to a permanent academic role thereafter.^7^, ^8^ Academic career progression in the animal health sector is also thought to be problematic, but has barely been investigated in the UK since a pioneering study undertaken 20 years ago exploring participants’ perceptions of their career choices.^9^, ^10^, ^11^ That study compared the characteristics of veterinarians who had pursued a research career with matched controls who had not worked in research, and reported low salary and job insecurity as key perceived disadvantages of a veterinary research career, demonstrating that problems in this sector are not new.^9^, ^10^, ^11^ However, quality research (and research‐informed teaching) that advances animal welfare requires a supply of skilled academic staff, making this issue highly relevant to stakeholders across the animal health sector, including research funders.

The current paper is part of a larger research project, the UK Canine Research Funding Analysis Project, which was carried out to improve the evidence base on canine research and its funding.^12^ Phase 1 of that project mapped the landscape of UK not‐for‐profit canine health and welfare research funding between 2012 and 2022, tracing funding patterns across donor and recipient organisations, investigating the support allocated to different fields of research and developing metrics to measure the canine benefit and impact of each research project.^13^ This phase identified £57.8 million of canine‐relevant research funding, supporting over 500 research projects. Overall, wide‐scope funders (UK Research and Innovation Councils and the Wellcome Trust) tended to support human‐directed research, much of it concerning One Health, while animal‐directed funders (UK charities and other not‐for‐profit funding organisations) tended to support research that was specifically canine directed. Phase 2 aimed to identify the highest priorities in UK canine health and welfare and its research and the highest priorities for future change in this sector.^14^ This investigation involved a modified Delphi study, which drew on the opinions and experiences of almost 60 sector stakeholders with wide‐ranging and varying expertise in canine health and welfare, with a roughly equal divide between veterinary professionals and other non‐veterinary professionals with relevant expertise. These participants identified a lack of opportunities and inadequate support for early career researchers (including both veterinary professionals and those with other prior experience) as two of the highest priority issues currently affecting canine health and welfare research infrastructure in the UK, highlighting the need for better understanding of these challenges.^14^ This Delphi study confirmed that the career difficulties faced both by veterinary professionals and by early career researchers are widely recognised as problems within the canine health and welfare sector, and therefore merit further investigation.

However, little is known about the current numbers, demography, training pathways or career outcomes of veterinary professionals and non‐veterinary professionals seeking to enter a career in animal‐related research more broadly, or the canine research sector in particular, mirroring a wider lack of data on the pathways followed by master's and PhD graduates across all fields, particularly those who leave academia.^7^ The current work aimed to address some of these critical data gaps by conducting a preliminary quantitative study to contribute some baseline information on patterns of student funding in canine health and welfare research, and the career outcomes of those in receipt of it. It draws on the canine research funding dataset described above to identify cohort characteristics and career outcomes (as of August 2024) for postgraduate students enrolled in master's or PhD research degree courses in canine health and welfare who received not‐for‐profit funding between 2012 and 2018. By describing this cohort, the current study aimed to inform the debate over early career challenges for veterinarians and researchers and to suggest areas that merit further consideration by funders and employers in this sector.

### Note on terminology

For ease of understanding and consistency, the term ‘student’ is used throughout to refer to the people in the dataset. The term ‘former student’ is sometimes used where this would aid clarity in discussing a particular point. No registered veterinary nurses were identified in the dataset. Therefore, for brevity, the terms ‘veterinarians’ and ‘non‐veterinarians’ are used throughout the paper to differentiate between veterinary professionals and those without a veterinary qualification.

## MATERIALS AND METHODS

### Dataset

This study drew on the dataset developed in the first phase of the overall research project, which has been described previously.^13^ This comprised total funding data for £57.8 million of canine‐relevant funding, provided by 10 not‐for‐profit wide‐scope funders (UK Research and Innovation Councils and the Wellcome Trust), 18 animal‐directed funders (UK charities and other not‐for‐profit funding organisations) and 81 breed community groups, and representing 684 funding awards supporting over 500 separate research projects made between 2012 and 2022. These data had already been tabulated, cleaned and organised in MS Excel to categorise each research project by previously described metrics.^13^

The current study analysed a subset of these previous data, comprising only those 452 awards made between 2012 and 2018. The 2018 cut‐off was chosen so that all grant holders had sufficient time to complete their studentships before the current study was conducted in August 2024, given that UK PhD studentships are usually 3 years but may be double this length if part‐time. These data were visually scrutinised to identify research grants that were specifically intended to support master's or PhD studentships or where project descriptions specified that a larger award included the support of a research studentship. Using the title and research institution of each project as search terms, each supported student was identified and the outcome of each research project was established through searching publicly available online university thesis repositories, university news bulletins and other data sources such as ResearchGate and published papers. Student gender was identified through name (supported by internet searches to identify the typical gender associated with that name, where appropriate), visual images, personal knowledge, where relevant, and self‐description, where available. Other demographic attributes were not assessed, apart from obtaining an approximate overview of student age (young adult/mature student). Where students could not be identified or there was no evidence that the research project had been completed, the awarded grant was excluded from the final analytic dataset. Using each supported student's name as a search term, their subsequent employment destinations (as of August 2024) were established by manually searching public online databases, chiefly LinkedIn, ResearchGate, the Royal College of Veterinary Surgeons’ online database, and relevant university websites. For academic positions, whether such roles were temporary or permanent was generally clear from the job title (e.g., postdoctoral researcher, professor). For non‐academic positions, it was sometimes less clear whether contracts were temporary or permanent, but in most cases, this could be inferred with reasonable certainty from individual circumstances, for example, from senior commercial job titles or for veterinary specialists who had moved into private practice.

### Data analysis

Using MS Excel, these data were cleaned and coded to categorise each individual student by a range of demographic and career‐related criteria, such as gender, type of postgraduate degree, and whether in permanent employment in 2024. Table 1 shows the full list of analytical categories used. Student research topics were categorised in two ways. Using previously described topic categories,^13^ the main species focus of each research project was identified, and each was recorded as a human‐focused project (research that investigated dogs primarily to improve human health, either in a One Health context or as a model for human disease) or an animal‐focused project (primarily concerned with the health and welfare of dogs, or of dogs and other animals such as cats). Student research topics were also categorised as ‘hands‐on’ or ‘hands‐off’ projects, according to the nature of the data collected in their project (some projects employed multiple or interdisciplinary methodologies). ‘Hands‐on’ approaches included studies based largely on clinical or physical measurements of actual dogs, studies based on observations of canine behaviour, and research that primarily involved laboratory‐based skills in subjects such as molecular genetics or cellular pathology. ‘Hands‐off’ approaches included projects in the humanities and social sciences, involving surveys, interviews or archival work, and data science projects, involving computer modelling or database manipulation.

**TABLE 1:** Data categories used in quantitative analysis, together with main options used for statistical analysis and explanatory comments, where relevant.

Variable	Main categories	Comments
Student ID	Number code	
Student gender	Male/female/other	Two categories used in analysis as no students were identified as other gender/s
Veterinary surgeon?	Yes/no	
Type of university where studentship held	UK veterinary school/other university (UK or international)	Universities without veterinary schools were differentiated from universities with veterinary schools/the RVC
Funder type	Wide scope/animal directed	Wide scope = Government and Wellcome Trust; animal directed = animal charities and similar funding organisations
Degree type	Research master's/PhD	
Research focus	Human focused/animal focused	Human‐focused research studies dogs in a One Health or human medicine context; animal‐focused research concerns dogs alone or together with other animal species
Degree main skillset	‘Hands‐on’/‘hands‐off’	‘Hands‐on’ research: clinical/physical observations, behavioural studies and laboratory‐based investigations; ‘hands‐off’ research: humanities, social science and data science
Permanent job?	Yes/no	Career outcomes ascertained through information on public websites
Currently working in academia?	Yes/no	
Currently working with animals?	Yes/no	
Working in clinical practice now?	Yes/no	Only applicable to veterinarians in the dataset

Abbreviation: RVC, Royal Veterinary College.

These data were then coded numerically and exported to SPSS Statistics version 29.0 (IBM) for statistical analysis. All data were anonymised for analysis by removing names and project titles from the data fields.

Descriptive statistics reported the frequency and percentage for relevant variables. Associations between demographic and career‐related variables were investigated using Pearson's chi‐square testing, with adjustment using continuity correction for 2 × 2 tables and Fisher's exact test where expected cell counts were less than 5.^15^ Statistical significance was set at *p*‐value of less than 0.05.

## RESULTS

The final dataset of the current study contained 50 identified postgraduate studentships funded between 2012 and 2018 inclusive from the 684 grants awarded in the original project dataset. The remaining 634 grants were excluded because 232 grants were awarded from 2019 onwards; 396 grants could not be traced as supporting a postgraduate student; one known master's or PhD project was abandoned; and for five known master's or PhD projects, the supported student could not be identified. The 2024 employment status could not be ascertained for four students (all were female non‐veterinarians), so the analysis of 2024 employment outcomes was based on a reduced dataset of 46 students.

### Student demographics

Of the 50 students analysed, 40 (80.0%) were female and 10 (20.0%) were male (no students were publicly recorded as non‐binary, gender neutral or other genders). Visual scrutiny of students’ online public profiles suggested that most had continued postgraduate education directly or soon after completing a veterinary degree or an undergraduate science degree as a young adult. Approximately one‐quarter of the cohort (*n* = 11, 22.0%) were research master's students and three‐quarters (*n* = 39, 78.0%) were PhD students. Just under half (*n* = 23, 46.0%) were qualified veterinarians. No registered veterinary nurses were identified in the dataset.

Tested associations between variables are shown in Table 2.

**TABLE 2:** Analytical categories and paired variables used in chi squared and Fisher's exact association testing, showing number and percentage of students in each paired group.

Analytical category	Association tested	Group 1	Group 2	Group 3	Group 4	Total *n* across groups	*p*‐Value
Demography and funding	Gender versus veterinarian	Female/veterinarian, *n* = 20 (40.0%)	Female/non‐veterinarian, *n* = 20 (40.0%)	Male/veterinarian, *n* = 3 (6.0%)	Male/non‐veterinarian, *n* = 7 (14.0%)	50	0.308
	Gender versus degree type	Female/master's, *n* = 9 (18.0%)	Female/PhD, *n* = 31 (62.0%)	Male/master's, *n* = 2 (4.0%)	Male/PhD, *n* = 8 (16.0%)	50	>0.999
	Veterinarian versus degree type	Veterinarian/master's, *n* = 7 (14.0%)	Veterinarian/PhD, *n* = 16 (32.0%)	Non‐veterinarian/master's, *n* = 4 (8.0%)	Non‐veterinarian/PhD, *n* = 23 (46.0%)	50	0.324
	Funder type versus veterinarian	Wide scope/veterinarian, *n* = 8 (16.0%)	Wide scope/non‐veterinarian, *n* = 12 (24.0%)	Animal directed/veterinarian, *n* = 15 (30.0%)	Animal directed/non‐veterinarian, *n* = 15 (30.0%)	50	0.685
	Funder type versus degree type	Wide scope/master's, *n* = 1 (2.0%)	Wide scope/PhD, *n* = 19 (38.0%)	Animal directed/master's, *n* = 10 (20.0%)	Animal directed/PhD, *n* = 20 (40.0%)	50	**0.033**
	Veterinarian versus university type	Veterinarian/veterinary school, *n* = 21 (42.0%)	Veterinary/not veterinary school, *n* = 2 (4.0%)	Non‐veterinary/veterinary school, *n* = 19 (38.0%)	Non‐veterinarian/not veterinary school, *n* = 8 (16.0%)	50	0.085
	Funder type versus university type	Wide scope/veterinarian school, *n* = 12 (24.0%)	Wide scope/not veterinary school, *n* = 8 (16.0%)	Animal directed/veterinary school, *n* = 28 (56.0%)	Animal directed/not veterinary school, *n* = 2 (4.0%)	50	**0.009**
Research project type	Research focus versus veterinarian	Human focused/veterinarian, *n* = 3 (6.0%)	Human focused/non‐veterinarian, *n* = 6 (12.0%)	Animal focused/veterinarian, *n* = 20 (40.0%)	Animal focused/non‐veterinarian, *n* = 21 (42.0%)	50	0.479
	Research focus versus university type	Human focused/veterinarian school, *n* = 3 (6.0%)	Human focused/not veterinary school, *n* = 6 (12.0%)	Animal focused/veterinary school, *n* = 37 (74.0%)	Animal focused/not veterinary school, *n* = 4 (8.0%)	50	**<0.001**
	Research focus versus funder type	Human focused/wide scope, *n* = 6 (12.0%)	Human focused/animal directed, *n* = 3 (6.0%)	Animal focused/wide scope, *n* = 14 (28.0%)	Animal focused/animal directed, *n* = 27 (54.0%)	50	0.130
	Project skillset versus veterinarian	‘Hands‐on’/veterinarian, *n* = 16 (32.0%)	‘Hands‐on’/non‐veterinarian, *n* = 17 (34.0%)	‘Hands‐off’/veterinarian, *n* = 7 (14.0%)	‘Hands‐off’/non‐veterinarian, *n* = 10 (20.0%)	50	0.848
Employment outcomes	Gender versus permanent job	Female/permanent job, *n* = 18 (39.1%)	Female/not permanent job, *n* = 18 (39.1%)	Male/permanent job, *n* = 5 (10.9%)	Male/not permanent job, *n* = 5 (10.9%)	46	>0.999
	Academia versus permanent job	In academia/permanent job, *n* = 9 (19.6%)	In academia/not permanent job, *n* = 20 (43.5%)	Not in academia/permanent job, *n* = 14 (30.4%)	Not in academia/not permanent job, *n* = 3 (6.5%)	46	**0.002**
	Veterinarian versus academia	Veterinarian/in academia, *n* = 12 (26.1%)	Veterinarian/not in academia, *n* = 11 (23.9%)	Non‐veterinarian/in academia, *n* = 17 (37.0%)	Non‐veterinarian/not in academia, *n* = 6 (13.0%)	46	0.222
	Veterinarian versus permanent job	Veterinarian/permanent job, *n* = 16 (34.8%)	Veterinarian/not permanent job, *n* = 7 (15.2%)	Non‐veterinarian/permanent job, *n* = 7 (15.2%)	Non‐veterinarian/not permanent job, *n* = 16 (34.8%)	46	**0.018**
	Veterinarian versus permanent job (clinical/practice roles excluded)	Veterinarian/permanent job, *n* = 8 (21.6%)	Veterinarian/not permanent job, *n* = 6 (16.2%)	Non‐veterinarian/permanent job, *n* = 7 (18.9%)	Non‐veterinarian/not permanent job, *n* = 16 (43.2%)	37	0.208
	Research focus versus permanent job	Human focused/permanent job, *n* = 2 (4.4%)	Human focused/not permanent job, *n* = 7 (15.2%)	Animal focused/permanent job, *n* = 21 (45.7%)	Animal focused/not permanent job, *n* = 16 (34.8%)	46	0.135
	Project skillset versus permanent job	‘Hands‐on’/permanent job, *n* = 20 (43.5%)	‘Hands‐on’/not permanent job, *n* = 10 (21.7%)	‘Hands‐off’/permanent job, *n* = 3 (6.5%)	‘Hands‐off’/not permanent job, *n* = 13 (28.3%)	46	**0.005**
	Project skillset versus academia	‘Hands‐on’/in academia, *n* = 17 (37.0%)	‘Hands‐on’/not in academia, *n* = 13 (28.3%)	‘Hands‐off’/in academia, *n* = 12 (26.1%)	‘Hands‐off’/not in academia, *n* = 4 (8.7%)	46	0.365
	Job focus versus academia	Academia/human‐focused job, *n* = 10 (21.7%)	Academia/animal‐focused job, *n* = 19 (41.3%)	Not in academia/human‐focused job, *n* = 3 (6.5%)	Not in academia/animal‐focused job, *n* = 14 (30.4%)	46	0.315
	Job focus versus permanent job	Human‐focused job/permanent job, *n* = 4 (8.7%)	Human‐focused job/not permanent job, *n* = 9 (19.6%)	Animal‐focused job/permanent job, *n* = 19 (41.3%)	Animal‐focused job/not permanent job, *n* = 14 (30.4%)	46	0.190
	Job focus versus research focus	Human‐focused degree/human‐focused job, *n* = 7 (15.2%)	Human‐focused degree/animal‐focused job, *n* = 2 (4.4%)	Animal‐focused degree/human‐focused job, *n* = 6 (13.0%)	Animal‐focused degree/animal‐focused job, *n* = 31 (67.4%)	46	**<0.0** **01**
	Job focus versus veterinarian	Veterinarian/human‐focused job, *n* = 1 (2.2%)	Veterinarian/animal‐focused job, *n* = 22 (47.8%)	Non‐veterinarian/human‐focused job, *n* = 12 (26.1%)	Non‐veterinarian/animal‐focused job, *n* = 11 (23.9%)	46	**0.001**
	Job focus versus veterinarian (currently clinical roles excluded)	Veterinarian/human‐focused job, *n* = 1 (2.7%)	Veterinarian/animal‐focused but non‐clinical job, *n* = 13 (35.1%)	Non‐veterinarian/human‐focused job, *n* = 12 (32.4%)	Non‐veterinarian/animal‐focused job, *n* = 11 (29.7%)	37	**0.011**

*p*‐values <0.05 shown in bold.

Gender was not significantly associated with holding a prior veterinary qualification (*p* = 0.308) or with whether the funding was for a master's or PhD degree (*p* > 0.999). Students with a prior veterinary qualification were not significantly more likely to be funded for either a master's or PhD degree (*p* = 0.324).

### Project trends

Of 50 students, wide‐scope funders supported 20 (40.0%) students and animal‐directed funders supported 30 (60.0%) students. There was no significant association between the proportion of veterinarians and non‐veterinarians supported by wide‐scope funders and animal‐directed funders (*p* = 0.685). However, wide‐scope funders were less likely to support master's students than animal‐directed funders, and more likely to support PhD students (*p* = 0.033).

The majority (*n* = 40, 80.0%) of funded students were based at UK universities with veterinary schools, but 10 (20.0%) studied at other UK or international universities or institutions. There was no significant difference between veterinarians and non‐veterinarians in their likelihood of studying at universities with or without veterinary schools (*p* = 0.085). However, students at veterinary schools were more likely to be supported by animal‐directed funders, while students at universities that did not include a veterinary school were more likely to be supported by wide‐scope funders (*p* = 0.009).

There was considerable variety in the types of research project conducted across these 50 studentships (Table 3). Most (82.0%) of the students studied broadly animal‐focused topics, with canine‐focused specific topics accounting for almost three‐quarters (74.0%) of all projects. Students at universities with a veterinary school were significantly more likely to study an animal‐focused topic than those at a university without a veterinary school (Table 2; *p *< 0.001), with 37 of 41 (90.0%) animal‐focused research projects undertaken at universities with a veterinary school. However, there was no statistically significant difference between wide‐scope funders and animal‐directed funders in supporting human‐focused or animal‐focused research (*p* = 0.130), or between veterinarians and non‐veterinarians in the likelihood of undertaking human‐focused or animal‐focused research (*p* = 0.479).

**TABLE 3:** Student research project types, showing broad categories and specific categories for research topic focus and degree main skillset.

Research project type	Broad category	Specific category
Research focus	Human focused, *n* = 9 (18.0%)	One Health/One Medicine, *n* = 7 (14.0%)
		Human medicine, *n* = 2 (4.0%)
	Animal focused, *n* = 41 (82.0%)	Canine focused, *n* = 37 (74.0%)
		Dogs and other animals, *n* = 4 (8.0%)
Total number of projects	50	50
Degree main skillset	‘Hands‐on’, *n* = 33 (66.0%)	Clinical/physical studies of dogs, *n* = 7 (14.0%)
		Canine behaviour, *n* = 4 (8.0%)
		Laboratory work, *n* = 22 (44.0%)
	‘Hands‐off’, *n* = 17 (34.0%)	Humanities and social science, *n* = 9 (18.0%)
		Data science, *n* = 8 (16.0%)
Total number of projects	50	50

Two‐thirds (66.0%) of all students conducted research projects with a primarily ‘hands‐on’ skillset, with almost half (44.0%) of all projects primarily involving laboratory‐based skills and about a fifth (22.0%) primarily involving direct clinical, physical or behavioural observations of actual dogs (Table 3). There was no statistically significant difference between veterinarians and non‐veterinarians in the likelihood of undertaking ‘hands‐on’ or ‘hands‐off’ projects, as shown in Table 2 (*p* = 0.848).

### Employment outcomes

Exactly half (*n* = 23, 50.0%) of traceable students were recorded as having obtained permanent jobs in 2024 (Table 2); however, as contract status could not always be firmly established, especially for roles outside academia, these data may contain some uncertainty. Gender was not significantly associated with the probability of obtaining a permanent job (*p* > 0.999). Among the 23 students who had not obtained permanent jobs, 22 were currently employed on short‐term research contracts or other short‐term contracts at universities or other organisations, and one was currently ‘looking for work’ on LinkedIn, having recently finished a postdoctoral research contract. Approximately two‐thirds of the former students were still working within academia in 2024 (*n* = 29, 63.0%). Those students currently working within academia were significantly less likely to have a permanent role than those working outside academia (*p* = 0.002). Holding a veterinary degree was not significantly associated with the likelihood of working within academia (*p* = 0.222). Non‐veterinarians were less likely to have secured a permanent job than veterinarians (*p* = 0.018). However, by 2024, nine of the veterinarians with permanent jobs had returned to veterinary work in clinical and/or specialist practice, for which they were eligible because of their prior veterinary qualifications (and, in some cases, additional board certification), rather than because of their postgraduate research qualifications. When these former students were removed from the dataset, there was no significant difference between veterinarians and non‐veterinarians in their likelihood of securing a permanent job after obtaining a research degree (*p* = 0.208).

The likelihood of securing a permanent job was not significantly associated with research degree focus (human focused or animal focused) (*p* = 0.135). In contrast, research project main skillset was strongly associated with the likelihood of subsequent permanent employment. ‘Hands‐off’ projects were less likely to lead to permanent employment than ‘hands‐on' projects (*p* = 0.005). However, students who had undertaken ‘hands‐off’ research projects were not significantly more likely to be employed within academia in 2024 than those who had undertaken ‘hands‐on’ research projects (*p* = 0.365).

Considering current (August 2024) employment, 33 of 46 (71.7%) students were working in animal‐focused roles, where advancing canine or other animal health and welfare was the primary aim; this included clinical and non‐clinical occupations of all types, such as private practice, research or charity work. The remaining 13 (28.3%) students were all employed in various human health‐focused or One Health‐focused roles, such as medical research or medical writing. Nobody whose current employment could be ascertained had left the science and health sector to work in an entirely unrelated field, and as noted above, only one person was identified as currently seeking work; thus, 45 of 46 (97.8%) students whose employment status could be ascertained were in health‐sector‐relevant work of some kind. There was no significant association between students working in animal‐focused roles and the likelihood of them working in academia (*p* = 0.315) or having a permanent job (*p* = 0.190). However, students who had previously researched animal‐focused topics were more likely to be working in animal‐focused roles, and students who had researched human‐focused topics were more likely to be working in human‐focused roles (*p* < 0.001). Moreover, veterinarians were more likely to be working in animal‐focused jobs and less likely to be working in human‐focused jobs than non‐veterinarians (*p* = 0.001). This difference remained statistically significant even when veterinarians currently working in clinical roles were excluded (*p* = 0.011).

## DISCUSSION

This study explored characteristics and career outcomes (as at 2024) of UK research degree students in canine health and welfare funded by UK not‐for‐profit organisations from 2012 to 2018. Almost all former students had secured science or health‐related employment: a majority had remained in academia, but these were less likely to have secured an apparently permanent job than their peers. Former students who were veterinarians and/or who undertook animal‐focused research were more likely to be working in animal‐focused jobs than non‐veterinarians or those who had undertaken human‐focused research.

Although this study was canine focused, some of the students also worked with other companion species, particularly cats, and so its findings are relevant to the wider small companion animal research sector, but potentially less so for the agricultural and equine spheres, where different factors may operate due to different research funder involvement and career pathways. However, we consider that dogs are likely to attract much more research funding than other small animal species. Canine research receives significant investment by wide‐scope funders (government research funding councils and similar organisations such as the Wellcome Trust) because of the significance of canine zoonotic disease and dogs’ use as a model for human diseases.^13^ Moreover, the largest two animal‐directed funders of canine research in the studied dataset (Dogs Trust and the Kennel Club Charitable Trust) only supported canine‐focused research. Other small companion animal species, such as the cat or rabbit, in our experience do not receive species‐specific support on a similar scale. Therefore, although the current study was limited to canine‐relevant research studentships, the results are likely to represent the majority of small companion animal student research funding at that time.

Postgraduate degrees that were self‐funded, internationally funded, funded by internal university budgets, funded by other means (such as commercial organisations) or that only concerned non‐canine species were intentionally excluded. Some canine‐relevant funded postgraduate research degrees will also be missing from the dataset, either as known omissions because the funded student could not be identified or as unknown omissions because the grant had not been identified originally. Nevertheless, because the original funding dataset was comprehensive, including 684 funding grants, it is likely that the dataset under investigation here does capture the majority of UK not‐for‐profit‐funded research degrees in canine health and welfare from 2012 to 2018. Most grants in the original dataset did not visibly support students, often because they concerned relatively small sums awarded to established researchers to carry out targeted projects investigating specific aspects of canine health and welfare (for example, molecular genetics investigation of an inherited disease in a certain breed) and, in some cases, because relatively large amounts were directed towards major technological initiatives such as establishing various new biological databases (for example, genomic libraries) rather than towards student‐accessible investigations.^13^

Since only 50 studentships were identified during this 7‐year period, this suggests that this field of research is relatively small, with an average of only about seven UK not‐for‐profit‐funded research degrees in canine health and welfare identified per year. The current study did not attempt to investigate how competitive this funding was to obtain, but inevitably the small number of funded roles annually suggests that this is a very restricted field that will offer few entry opportunities if compared, for example, to the 1269 new registrants with the RCVS in 2021, over half of whom are likely to enter small animal practice.^1^ Other students in this field may have been funded by commercial companies through major grants that do not separate student funding in an identifiable way or, in a very few cases, self‐funded, but identifying such students would require a separate investigation.

The cut‐off date of 2018 was chosen to ensure that sufficient time had elapsed since funding was awarded to permit the students to have completed their degrees. Since the cohort was defined by having received a funded research studentship, all included students had necessarily undertaken research degrees. Nevertheless, there was unavoidable heterogeneity in the cohort according to how long each degree would have taken (e.g., master's vs. PhD, part‐time vs. full‐time, possible delays due to COVID‐19 or other factors for some students) and when it commenced. Consequently, a snapshot of career destinations in August 2024 will potentially have included employment up to 10 years after graduation for a 2012 master's student alongside employment around the time of completion for a 2018 PhD student who took 5 years to complete their degree. However, given the heterogeneity of the cohort's career pathways in other ways, this unavoidable variation can be regarded as a study limitation rather than an invalidation.

Because this was a retrospective study based on publicly available human data, it was not possible to conduct a detailed demographic analysis. However, the gender bias towards women (80% female/20% male) in the study dataset mirrors the 78% of respondents who identified as female in the first (survey) round of the modified Delphi study previously conducted within this research project,^14^ and is consistent with previously reported gender ratios within the veterinary sector.^16^ Although it was not possible to accurately document the ages of students in the dataset, it was apparent from online information that the majority were likely to have entered postgraduate education as relatively young adults. However, a substantial minority (mostly veterinarians) had evidently undertaken funded postgraduate study after some years in the workplace, and for these older students, career pathways showed considerable individuality. Further qualitative research would be necessary to explore the career motivations of these people and establish any underlying patterns.

This study supports previous findings that reported marked differences in other funding patterns between wide‐scope funders and animal‐directed funders.^13^ In particular, wide‐scope funders were significantly less likely than animal‐directed funders to support master's degrees. Given that a master's degree is frequently a prerequisite for acceptance of a PhD programme and that cost is a major barrier to undertake a master's degree,^17^ sector‐specific support from animal‐directed funders is therefore critical for facilitating entry into research, particularly for students with additional barriers to access. In contrast, wide‐scope funders were significantly more likely to support PhD students than animal‐directed funders, suggesting that prospective PhD students or principal investigators may find it advantageous to develop a project that is eligible for wide‐scope funding, where this is feasible.

Students at veterinary schools were more likely to be supported by animal‐directed funders and more likely to be studying an animal‐focused topic than students at universities without a veterinary school. This association is unsurprising, since previous research has shown that almost three‐quarters of UK canine‐focused research funding is provided by animal‐directed funders^13^ and that animal‐focused health expertise is institutionally concentrated in veterinary schools. In contrast, however, comparison between veterinarians and non‐veterinarians revealed few differences. There was no significant difference between the two groups in their study of human‐focused versus animal‐focused topics or of degrees centring ‘hands‐off’ or ‘hands‐on’ research skillsets; veterinarians were as likely as non‐veterinarians to conduct research primarily involving remote data analysis rather than work that depended on contact with animals or technical laboratory investigations. These findings support the widespread viewpoint that a veterinary degree is a versatile qualification that can lead to many subsequent fields of work.^18^, ^19^

Almost two‐thirds (63.1%) of the overall studied cohort were still working in academia in 2024, a slightly higher proportion than may be typical for PhD graduates in general, where one study showed that only 43% remained in academia by 6 months after graduation.^7^ Moreover, this study confirms anecdotal evidence and industry assumptions in demonstrating that those currently working in academia were significantly less likely to have secured a permanent role in 2024 than those who had left the sector: pursuing an academic career does entail more precarious employment, at least in the short to mid‐term. It was particularly interesting to find that, although veterinarians were more likely than non‐veterinarians to have secured an apparently permanent role by 2024 (*p* = 0.018), this difference was largely accounted for by those who returned to work in more senior roles in clinical and/or specialist practice. These roles were inaccessible to non‐veterinarians, but did not mandatorily require a postgraduate research degree, although research degrees may have conferred a competitive advantage in the veterinary sector, and some of these clinicians were working in specialist clinical roles within university schools. The finding that veterinarians who cease clinical work to follow a research pathway then have similar permanent employment outcomes to non‐veterinarians suggests that veterinarians may not secure a competitive advantage within academia from their prior professional qualification, although they may nevertheless have drawn on that qualification in their subsequent careers and may find their new employment more congenial.

The finding that students who had undertaken ‘hands‐off’ data‐driven research were less likely to have secured permanent employment than those who had undertaken ‘hands‐on’ research invites further scrutiny. All eight of the prior students whose degrees had primarily involved data science remained in academia in 2024, and seven of eight were on short‐term postdoctoral contracts or research fellowships. This tolerance of uncertainty may reflect student preferences to remain in academia and/or in a chosen canine‐relevant field, given that the UK Government states that there is an unmet demand for data science skills in the wider workplace, where permanent contracts and higher salaries may be more achievable than in the animal health sector.^20^ The ‘hands‐off’ students who had studied humanities and social science showed a wider range of 2024 outcomes, with four of nine on short‐term postdoctoral contracts or research fellowships within academia, and four having secured other roles outside academia (one was ‘looking for work’), where they drew on their enhanced skillsets in various ways. In contrast, among the ‘hands‐on’ group, 14 of the 22 students who had conducted laboratory‐based research and five of the seven students who had conducted clinical research had secured apparently permanent roles of widely various types, both within and outside academia. These patterns could be explored further through qualitative studies, as they probably encompass a wide range of career goals and choices, and will be influenced by limitations such as geographical flexibility, especially for those pursuing successive short‐term academic contracts. They may nevertheless reflect previously documented research employability and career pathway differences between humanities and social science doctorates and those in STEM, which have been found to afford more research opportunities and lead to more career satisfaction, particularly if obtained at more prestigious institutions.^7^

Students who researched animal‐focused topics were much more likely to continue to animal‐focused jobs while students who researched human‐focused topics were more likely to continue to human‐focused jobs. This may be because research training is likely to have led to subsequent work in a related field, and because these patterns are likely to reflect prior underlying as well as newly acquired personal interests. This is supported by the finding that veterinarians with research degrees were significantly more likely to be working in animal‐focused jobs than non‐veterinarians, even when those currently employed in clinical roles were excluded. All but one of the 13 human‐focused positions identified within the dataset, which mostly concerned work in laboratory science, public health or epidemiology, were held by non‐veterinarians. It is likely that some of the human‐focused canine‐relevant research conducted by non‐veterinarians involved people who were primarily interested in human health and only incidentally studying dogs, whereas the veterinarians and non‐veterinarians who studied animal‐focused topics were pursuing a prior interest in dogs, accounting for this association.

Overall, all but one of the students whose employment status could be ascertained were working in a field that was linked to their research degree in some way (one ‘looking for work’ at the time of the study). These roles were hugely varied, encompassing a wide range of teaching and research roles in academia, clinical practice, employment at canine welfare organisations, work in scientific publishing and many other destinations. People who obtain a research degree may be strongly motivated to use their skills afterwards, explaining why nobody was identified as having left the health and science sector altogether.

The scope, focus and methodology of the current study markedly differed from those used in the previously mentioned study that compared UK veterinarians who pursued research careers to those following other pathways, conducted in 2005.^10^, ^11^ Therefore, limited direct comparisons can be drawn between the two studies.^10^, ^11^ Gender patterns differ between the two studies. In 2005, female veterinarians were less likely than males to pursue a career in research.^10^, ^11^ In contrast, gender ratios in the current study broadly mirror those in the wider sector, suggesting that this difference has been reduced. The 2005 study was commissioned due to concern that too few veterinarians wanted to enter research,^10^, ^11^ whereas the current study suggests that in 2024 very few funded research opportunities existed, at least within the canine sector. However, in 2005, the main reasons that veterinarians gave for leaving research were lack of funding opportunities and lack of job security^10^: despite its different methodology, the current study confirms that a career in academia still leads to more precarious employment than other alternatives. Despite the intellectual stimulation of a career in academia, reported as the most common reason for choosing a research pathway in 2005^10^ and anecdotally still important to potential researchers today, those considering this route should thus be aware of its difficulties. As is suggested for the wider PhD sector, there is a strong argument that research degrees in the canine sector should be designed to equip students for a subsequent career that may well be outside academia.^7^

This quantitative study could not explore students’ own reflections on their postgraduate studies. However, the fact that so many of this cohort continued to work in fields related to their studies suggests that canine‐relevant postgraduate research can lead to a range of interesting, if not necessarily lucrative or secure, future career opportunities. Graduates may derive significant value from this pathway, besides contributing to the advancement of canine health as expected by their research funders.^21^

## CONCLUSION

The 50 master's and PhD students in this study investigated a wide range of human‐focused and animal‐focused research topics. Almost all these students were later employed in a wide range of roles within the science and health sector, with two‐thirds remaining in academia at the time of the study. However, those remaining in academia were significantly less likely to have obtained a permanent job than those who had left academia, confirming the widespread perception that those seeking an academic career currently face considerable barriers to progression even after they have successfully obtained postgraduate research degrees. Further qualitative research that explored postgraduate student researchers’ expectations, experiences and outcomes, potentially broadened beyond the canine and/or veterinary sectors, could provide more insight to inform future research funding and policy creation.

## AUTHOR CONTRIBUTIONS

All three authors contributed substantially to the conception and design of this study and approved the final version for publication. Data acquisition, analysis and interpretation were carried out by Alison M. Skipper, who also drafted the manuscript. The manuscript was critically revised for important intellectual content by Dan G. O'Neill and Rowena M.A. Packer.

## CONFLICTS OF INTEREST

The authors declare they have no conflicts of interest.

## ETHICS STATEMENT

This retrospective study used anonymised research funding data and anonymised human personal data in the public domain. There were no direct animal or human participants. It has been approved by the Social Science Research Ethical Review Board at the Royal Veterinary College (URN SR2025‐01090508). A completed STROBE checklist was provided to the publishing journal.

## Data Availability

This research involved two types of data: anonymised research funding data and anonymised human personal data in the public domain. The research funding data were originally collected for use in previously published research (see https://doi.org/10.1371/journal.pone.0303498). Some of these harvested research funding data are in the public domain and have been uploaded to Figshare. It can be found here: https://doi.org/10.6084/m9.figshare.25353301. Other research funding data were shared in confidence with the researchers by third‐party funding organisations and are not in the public domain. Interested parties should contact the lead author for more information about these data. The human personal data used in this research are not available because they have been anonymised as a condition of ethical approval (see above for details).
